# Targeting ovarian cancer stem cells: a new way out

**DOI:** 10.1186/s13287-023-03244-4

**Published:** 2023-02-14

**Authors:** Huiying Ma, Tian Tian, Zhumei Cui

**Affiliations:** Department of Gynecology, The Affiliated Hospital of Qingdao University, Qingdao, People’s Republic of China

**Keywords:** Ovarian cancer, Cancer stem cells, Therapy

## Abstract

Ovarian cancer (OC) is the most lethal gynecological malignancy due to tumor heterogeneity, the lack of reliable early diagnosis methods and the high incidence of chemoresistant recurrent disease. Although there are developments in chemotherapies and surgical techniques to improve the overall survival of OC patients, the 5-year survival of advanced OC patients is still low. To improve the prognosis of OC patients, it is important to search for novel therapeutic approaches. Cancer stem cells (CSCs) are a subpopulation of tumor cells that participate in tumor growth, metastasis and chemoresistance. It is important to study the role of CSCs in a highly heterogeneous disease such as OC, which may be significant to a better understanding of the oncogenetic and metastatic pathways of the disease and to develop novel strategies against its progression and platinum resistance. Here, we summarized the current findings about targeting methods against ovarian cancer stem cells, including related signaling pathways, markers and drugs, to better manage OC patients using CSC-based therapeutic strategies.

## Introduction

Ovarian cancer (OC) is the fifth most common female cancer and the leading cause of mortality among gynecological tumors. In 2020, there will be approximately 221,750 new cases of OC diagnosed and 13,940 ovarian cancer deaths in the USA [1]. OC is a heterogeneous group of malignancies caused by multiple etiologies. Epithelial ovarian cancers (EOC) are accounting for 90% of OC. According to the new criteria by The World Health Organization (WHO) in 2014, EOC are divided to several morphological categories depending on cell type: high-grade serous (HGSC), low-grade serous (LGSC), mucinous carcinomas (MC), endometrioid carcinomas (EC), and clear-cell carcinomas (CCC) [2]. Except for WHO classification, there are 8 dichotomies for EOC: high grade versus low grade, ovarian versus extraovarian primary, extrauterine versus uterine primary, sporadic versus hereditary, orthodox versus alternative histology, p53 overexpression versus complete absence of immunophenotype, *TP53*-mutated versus intact precursor, and therapy responsive versus refractory. A better understanding of different dichotomies and molecular classifications is significant for the individual diagnosis and therapy for EOC [3].

Despite intensive investigation, the pathogenesis of OC is not completely understood. HGSC arises from the ovarian surface epithelium or from the fallopian tube and LGSC arises from fallopian tube. Endometrioid carcinoma and clear cell carcinoma arise from endometriosis. Mucinous carcinoma arises from germ cells [4].

The high mortality rate is partly because many cases are diagnosed in advanced stages. In the early stage of OC, there are no obvious symptoms or efficient screening means; thus, early diagnosis is difficult. Over the past decades, the standard treatment for OC has been the combination of radical surgery and platinum-based chemotherapy [5].Although OC patients often initially respond to platinum/paclitaxel-based chemotherapy, most of them still experience relapses and eventually develop chemoresistance [6]. There are several different inhibitors targeting different cancer signal pathways, such as tyrosine kinase inhibitors and monoclonal antibodies, which exert antitumor role in affecting angiogenesis, cell growth and metastasis. Among these emerging inhibitors, the most promising one is poly-ADP-ribose polymerase inhibitors (PARPi), with greater benefit observed in patients with BRCA mutated or BRCA wild-type homologous recombination-deficient (HRD) tumors. According to the recent studies, there are new therapy strategies in HRD tumors, like combined PARPi with antiangiogenic agents, retreated with PARPis after previous PARPi treatment—“PARPi after PARPi” [7, 8]. The therapeutic effects of PARPi are limited, as treatment benefits only show on extending survival by a few months but not long-term survival [9]. Except for these, immunotherapy has also rapidly developed. Although treatment with immune checkpoint inhibitors, chimeric antigen receptor engineered T cells is rapidly developing, immunotherapy response rates among OC patients remain modest [10]. A number of researches discussed other novel therapeutic methods, such as agents targeting metabolism and biosynthetic pathways in OC cells, antibody drug conjugates targeting OC surface molecules and some new compounds whose mechanisms are not yet fully understood. Several studies focused on the gene mutations, like missense mutations in *p53* and mutations in *ARID1A* [11]. In spite of multiple therapy, most of OC patients eventually relapse and the 5-year survival rate for OC is less than 35% [12].

**Fig. 1:**
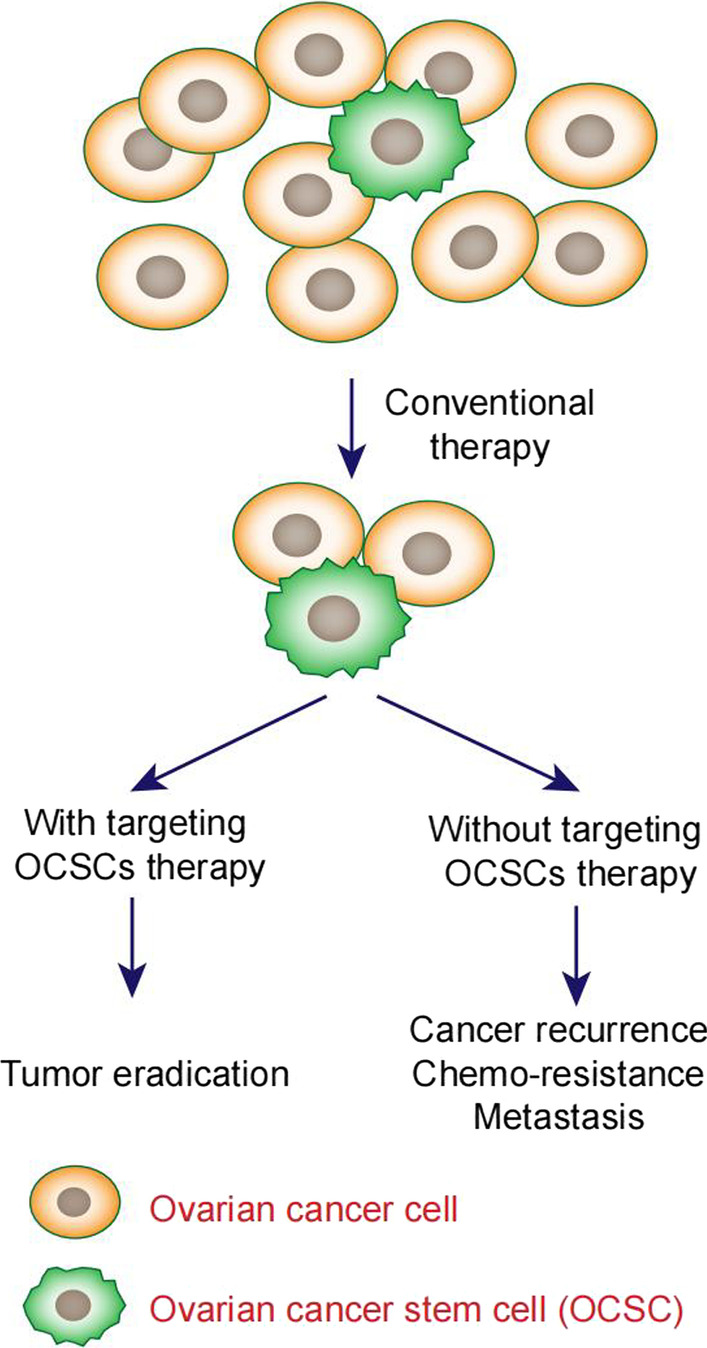
The properties of CSCs

There are three main models to explain histological and molecular heterogeneity for most solid tumors: the clonal evolution or stochastic model, the cancer stem cells (CSCs) or hierarchical model and plasticity model linking the two models above [13]. CSCs are a minor population of tumor cells with self-renewal, pluripotency and limitless proliferative properties [14]. The properties of CSCs are shown in Fig. 1. The CSC model suggests that regardless of the cell origins, many cancers have the same hierarchical organization as normal tissues and CSCs have similar molecular properties as normal stem cells. This model also indicates that the same CSC populations can originate from different cancer subtypes and the frequency of CSCs vary greatly, resulting in tumor heterogeneity [13]. CSCs have been identified in hematologic and solid cancers [15–19]. Experimental evidence supporting the existence of ovarian CSCs was reported by Bapat in 2005 [20]. They cultured a single clone from ascites capable of tumorigenesis and tumor differentiation. CSCs express distinct cell surface markers, such as CD24, CD44, and CD133 [21–23]. CSCs secrete proinflammatory cytokines and chemokines such as IL-4, IL-6, and IL8, contributing to self-renewal ability [24, 25]. However, the mechanism of how ovarian CSCs generate and how cell surface markers function are not known.

Previous studies reported that CSCs play an important role not only in tumorigenesis but also in tumor metastasis and tumor relapses due to resistance to chemotherapy and radiotherapy [26, 27]. Therefore, targeting ovarian CSCs may offer a novel therapeutic strategy to overcome the present dilemma. The elimination of ovarian CSCs is challenging partly due to heterogeneity, resulting in limitations in therapeutic efficacy. Herein, we provide a review of primary research on targeted therapy for ovarian cancer stem cells (OCSCs).

## Methods

The search strategy followed the methodological framework developed by Arksey and O’Malley, which was further clarified by Levac et al. and Joanna Briggs Institute [28–30]. Our review was conducted in five broad stages, each of which is outlined below.

1. Identifying the research questions

The central research question was “What is known from the existing literature about targeted therapy toward ovarian cancer stem cells?” As of this writing, multiple reviews have been published. We aim to gain a further understanding of all existing studies on ovarian CSCs through an analysis of published literature.

2. Identifying and selecting relevant studies

Final terms were determined after an initial broad search using PubMed, which was used to identify MeSH headings and free words used in relevant papers. We searched four electronic databases (PubMed, Web of Science, ScienceDirect and Embase) with date limitations between 2000 and 2022 for relevant articles. Both MeSH terms and free words texts were used. Table 1 provides the inclusion criteria and PubMed search strategy. Equivalent search strings were used in the other databases.

**Table 1:** Inclusion criteria and search strategy

Inclusion criteria	• Written in English
	• Articles that included study of both OC and CSCs in therapeutic aspect
	• Study date 2000 to 2022
Exclusion criteria	Review articles, case reports, conference reports, letters, editorial comments, opinions and non-English articles; studies of benign diseases, studies referring only to CSCs
Search strategy in PubMed	(("Ovarian Neoplasms" [Mesh]) OR ((((((((((((((((Neoplasm, Ovarian [Title/Abstract]) OR (Ovarian Neoplasm [Title/Abstract])) OR (Ovary Neoplasms [Title/Abstract])) OR (Neoplasm, Ovary [Title/Abstract])) OR (Neoplasms, Ovary [Title/Abstract])) OR (Ovary Neoplasm [Title/Abstract])) OR (Neoplasms, Ovarian [Title/Abstract])) OR (Ovary Cancer [Title/Abstract])) OR (Cancer, Ovary [Title/Abstract])) OR (Cancers, Ovary [Title/Abstract])) OR (Ovarian Cancer [Title/Abstract])) OR (Cancer, Ovarian [Title/Abstract])) OR (Cancers, Ovarian [Title/Abstract])) OR (Ovarian Cancers [Title/Abstract])) OR (Cancer of Ovary [Title/Abstract])) OR (Cancer of the Ovary [Title/Abstract]))) AND (("Stem Cells" [Mesh]) OR (((((((((((((((Cell, Stem [Title/Abstract]) OR (Cells, Stem [Title/Abstract])) OR (Stem Cell [Title/Abstract])) OR (Progenitor Cells [Title/Abstract])) OR (Cell, Progenitor [Title/Abstract])) OR (Cells, Progenitor [Title/Abstract])) OR (Progenitor Cell [Title/Abstract])) OR (Mother Cells [Title/Abstract])) OR (Cell, Mother [Title/Abstract])) OR (Cells, Mother [Title/Abstract])) OR (Mother Cell [Title/Abstract])) OR (Colony-Forming Unit [Title/Abstract])) OR (Colony Forming Unit [Title/Abstract])) OR (Colony-Forming Units [Title/Abstract])) OR (Colony Forming Units [Title/Abstract])))

3. Charting the data

We recorded the following data from the selected studies on a data extraction sheet: authors, year of publication, aims of the study, study type, study design and study outcomes.

4. Collating, summarizing and reporting the results

The selected articles were analyzed to address the research question. The data were collated into a summary of the study outcomes reported in narrative form.

## Results and discussion

1. Overview of included studies

**Fig. 2:**
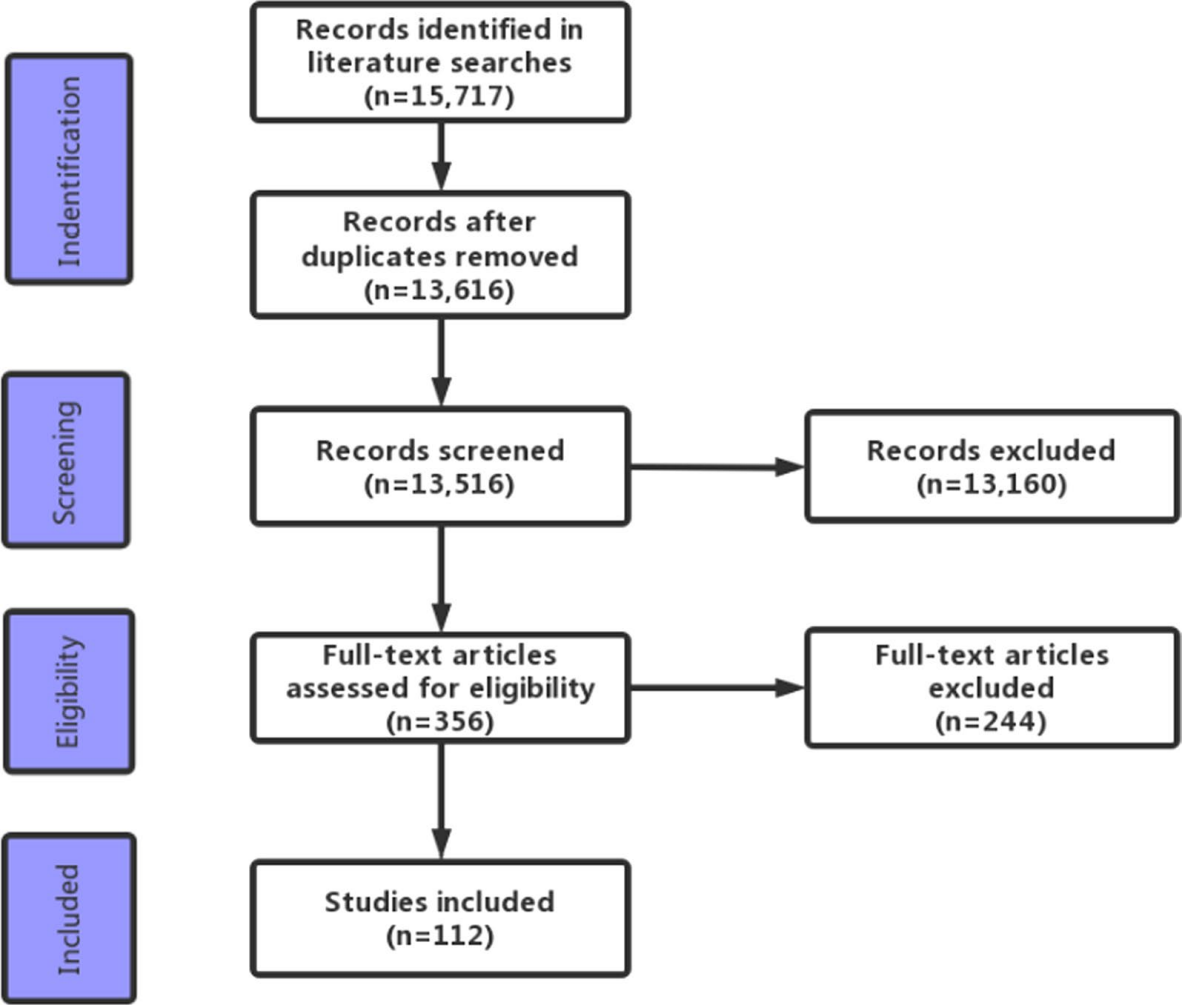
PRISMA flow diagram for review data evaluation

Initially, 15,717 scientific articles were found. After duplicate removal, 13,616 articles remained. A total of 13,160 articles were excluded after evaluation of the title and abstract. Two hundred forty further studies were excluded following full text review. As a result, 112 articles were included in the present review. Figure 2 shows the process of selecting studies. In Table 2, the relevant findings from some of included studies are summarized. In Table 3, the therapies targeting OCSCs showed in this review are summarized.

**Table 2:** Characteristics and major findings of included studies

Authors	Years	Targeting point or drugs	Major findings
Li [35]	2011	Notch pathway	Inhibition pf Notch signaling with the γ-secretase inhibitor DAPT inhibits OCSCs from self-renewing and proliferating
Shannon [32]	2012	Notch pathway	Notch3 siRNA knockdown depletes OCSCs and increases tumor sensitivity to platinum
Sham [36]	2014	Notch pathway	Withaferin A used alone or in combination with cisplatin targets putative OCSCs
Hyeok [37]	2016	Notch pathway	Galectin-3 maintains OCSCs by activating the Notch1 intracellular domain
Eun [129]	2016	Notch pathway	Hypoxia-Notch*-SOX2* signaling axis is vital for activation of OCSCs
Zhu [130]	2019	Notch 1-c-Myc pathway	SNORD89 regulates Notch 1-c-Myc pathway to promote cell stemness and acts as an oncogene in ovarian tumorigenesis
Zhang [131]	2020	Notch 1-c-Myc pathway	Stemness transformation of OC cells can be activated by SNORA72 through the Notch 1-c-Myc pathway
Chau [43]	2013	Wnt/β-catenin pathway	The activation of Wnt/β-catenin and ATP-binding cassette G2 downstream of c-Kit could promote OCSCs
Mariya [132]	2016	Wnt/β-catenin pathway	MMP10 activate canonical Wnt signaling by inhibiting noncanonical Wnt signaling ligand Wnt5a
Chen [133]	2017	Wnt/β-catenin pathway	STAT3 signaling maintained stemness and interconnected Wnt/β-catenin signaling via the miR-92a/DKK1 regulatory pathways
Deng [45]	2017	Wnt/β-catenin pathway	Rb1 and compound K can chemosensitize OCSCs, inducing a synergistic cytotoxicity via Wnt/β-catenin pathway and epithelial-to-mesenchymal (EMT) transition regulation
Wen [114]	2017	Wnt/β-catenin pathway	*SOX2* may directly bind to bcatenin and activate the Wnt/β-catenin pathway to maintain the stemness of ovarian spheroids
Srivastava [46]	2018	Wnt/β-catenin pathway	Calcitriol is able to deplete the OCSC population by inhibiting their Wnt signaling Pathway
Wu [134]	2015	Wnt/β-catenin pathway	MiR-1207 enhances the stem cell-like traits of OC cells by downregulating of multiple negative modulators of the Wnt/β-catenin pathway
Pan [44]	2018	Wnt/β-catenin pathway	Theaflavin-3, 3′-digallate inhibits OCSCs through Wnt/β-catenin signaling pathway
Deng [50]	2019	PI3K/Akt/mTOR pathway	EMT and enhanced CSC marker expression triggered by activated PI3K/Akt/mTOR signaling are involved in the chemoresistance of EOC
Ning [50, 52]	2016	PI3K/Akt/mTOR pathway	The reduced OVCSLC oncogenicity by DFOG through inhibitory effects on AKT and/or ERK and/or NF-κB pathways requires both FoxO3a and FoxM1 expression
Leizer [52]	2010	NF-κB pathway	EriB can inhibit NF-κB activity by down-regulating the level of nuclear p65
Jiang [52]	2022	NF-κB pathway	PFKFB3 is a key process of glycolysis in OCSCs and its inhibitor can impede stem properties
Xia [52]	2014	Hippo pathway	The self-renewal and chemoresistance properties of OCSCs depend on YAP and TEADs
Feng [52]	2016	Hippo pathway	VP can increase YAP expression, resulting in the suppression of OCSCs progression
Casagrande [71]	2011	CD44	Intraperitoneal CPE administration could eradicate CD44 + OCSCs after conventional therapy
Cheng [72]	2011	CD44	MiR-199a targets CD44 and reduces the proliferation and invasion of CD44 +/CD117 + ovarian cancer stem cells in vitro and in vivo
Wu [135]	2015	CD44	Human SKOV3 CD117 + CD44 + CSC-based vaccine may induce the anti-OCimmunity against tumor growth by reducing the CD117 + CD44 + CSC population
Skubitz [78]	2013	CD133	dCD133KDEL is a novel deimmunized toxin that appears to be targeting and eliminating CD133 + tumor initiating cells
Xiang [80]	2013	CD133	The stimulation function of IL-17 on self-renewal of CD133 + OCSCs might be mediated by NF-kB and MAPK signaling pathway
Wang [81]	2016	CD133	IL-23 contribute to ovarian cancer malignancy through promoting the self-renewal of CD133 + ovarian cancer stem-like cells
Long [79]	2016	CD133	The Cre/LoxP system-mediated tBid overexpression activated the pro-apoptotic signaling pathway and augmented the cytotoxic effect of cisplatin in CD133 + OCSCs
Kim [91]	2018	ALDH	ATRA suppressed ALDH1 expression, inhibiting NRF2 activation, which led to the attenuation of CSC-like properties in ALDH-H cells but not in ALDH-L cells
Choi [96]	2015	ALDH	BMP2 promotes the expansion of ALDH + C133 + OCSCs and restricting the growth of progenitor
Kakar [94]	2017	ALDH	WFA alone or when combined with CIS resulted in a significant suppression of tumorigenic function of isolated ALDH1 positive cancer stem cells in vitro
Young [92]	2014	ALDH	ATRA downregulates ALDH1/FoxM1/Notch1 signaling and suppresses tumor formation in OC cells
Cui [93]	2018	ALDH	DDB2, a transcription repressor, can abrogate ovarian CSC properties by downregulating ALDH1A1 expression
Shank [103]	2012	Metformin	Metformin can restrict the growth and proliferation of ovarian cancer stem cells in vitro and in vivo
Zhang [106]	2015	Metformin	Metformin at low dose inhibits selectively CD44 + CD117 + OCSCs through inhibition of EMT and potentiates the effect of cisplatin
Lee [115]	2017	Salinomycin	Combining salinomycin with other anti-cancer therapeutic agents can target OCSCs
Mi [116]	2017	Salinomycin	Salinomycin-loaded poly(lactic-co-glycolic acid)-poly(ethylene glycol) nanoparticles conjugated with CD133 antibodies can eliminate CD133 + OCSCs
Lee [123]	2020	Calcium channel blockers	Combination CCBs with cisplatni can inhibit the viability and proliferation of OCSCs
Lee [124]	2020	Calcium channel blockers	Poziotinib with a CCB can effectively inhibit OCSC survival and function

**Table 3:** Therapeutics targeting the OCSCs

Agent	Target	Mechanism of action	Study phase
DAPT	Notch pathway	GSIs	In vitro
WFA	Notch pathway	Notch1 inhibitor	Both in vitro and in vivo
Eugenol	Notch pathway	Notch-Hes1 inhibitor	Both in vitro and in vivo
TF3	Wnt/β-Catenin	β-Catenin down-regulator	In vitro
Ginsenoside-Rb1 metabolite compound K	Wnt/β-Catenin	β-Catenin down-regulator	Both in vitro and in vivo
Calcitriol	Wnt/β-Catenin	VDR down-regulator	Clinical trial phase 1
N-t-boc-Daidzein	PI3K/Akt/mTOR	Akt/mTOR down-regulator	In vitro
DFOG	PI3K/Akt/mTOR	FOXM1 inhibitor	In vitro
EriB	NF-κB	Nuclear p54 inhibitor	In vitro
3PO	NF-κB	PFKFB3 inhibitor	In vitro
PFK158	NF-κB	PFKFB3 inhibitor	Clinical trial phase 1
VP	Hippo	YAP inhibitor	Both in vitro and in vivo
dCD133KDEL	CD133	CD133 monoclonal antibody	Both in vitro and in vivo
ATRA	ALDH	ALDH1 inhibitor	Both in vitro and in vivo
DDB2	ALDH	ALDH1a1 inhibitor	Both in vitro and in vivo
Metformin	multiple	CD44 down-regulator	Clinical trial phase 2
Salinomycin	multiple	Apoptotic proteins promoter	Both in vitro and in vivo
CCBs	AKT/ERK pathway	Apoptotic proteins promoter	In vitro

2. OCSCs-related signaling pathways

**Fig. 3:**
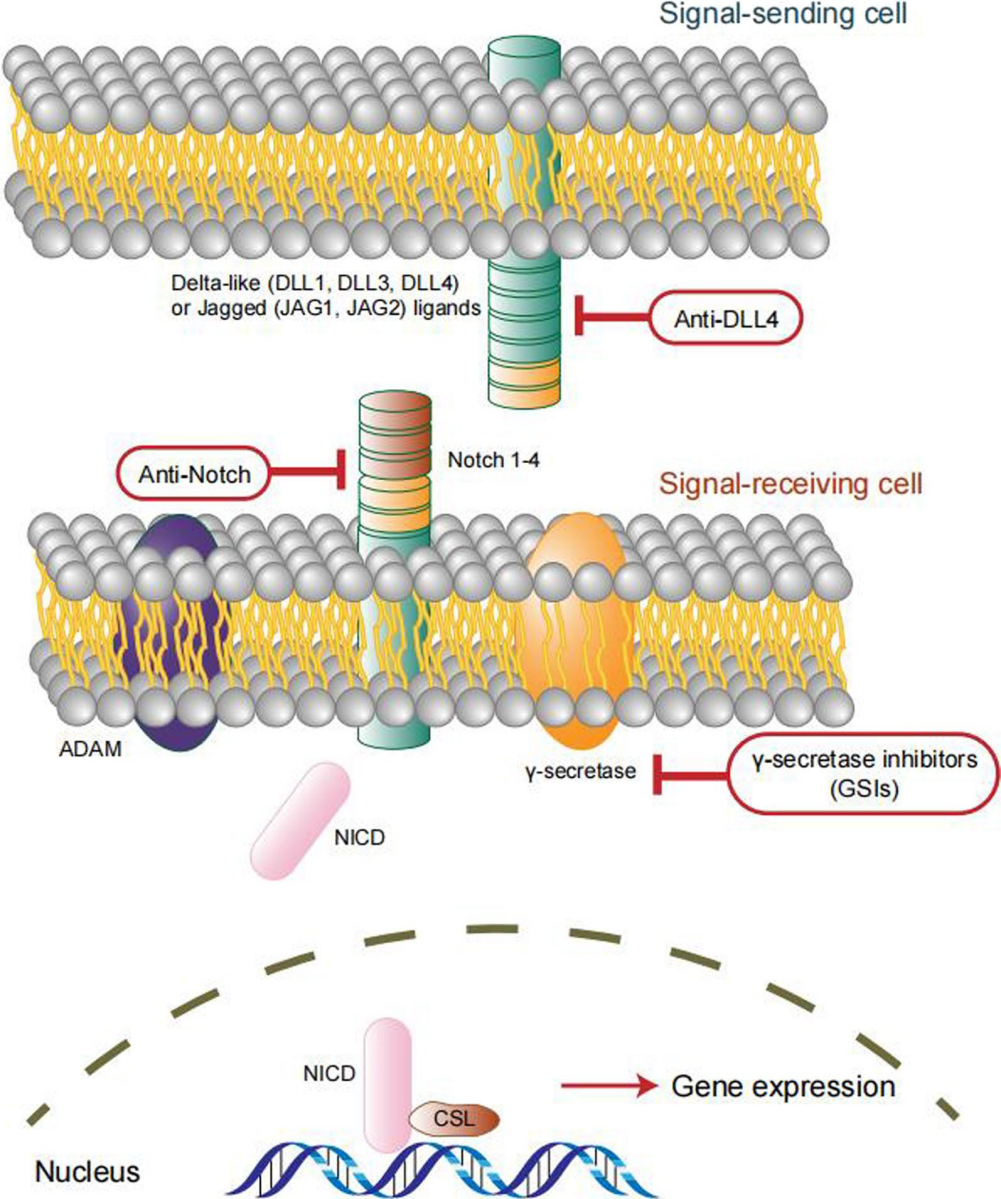
Notch signaling pathway in OCSCs

**Fig. 4:**
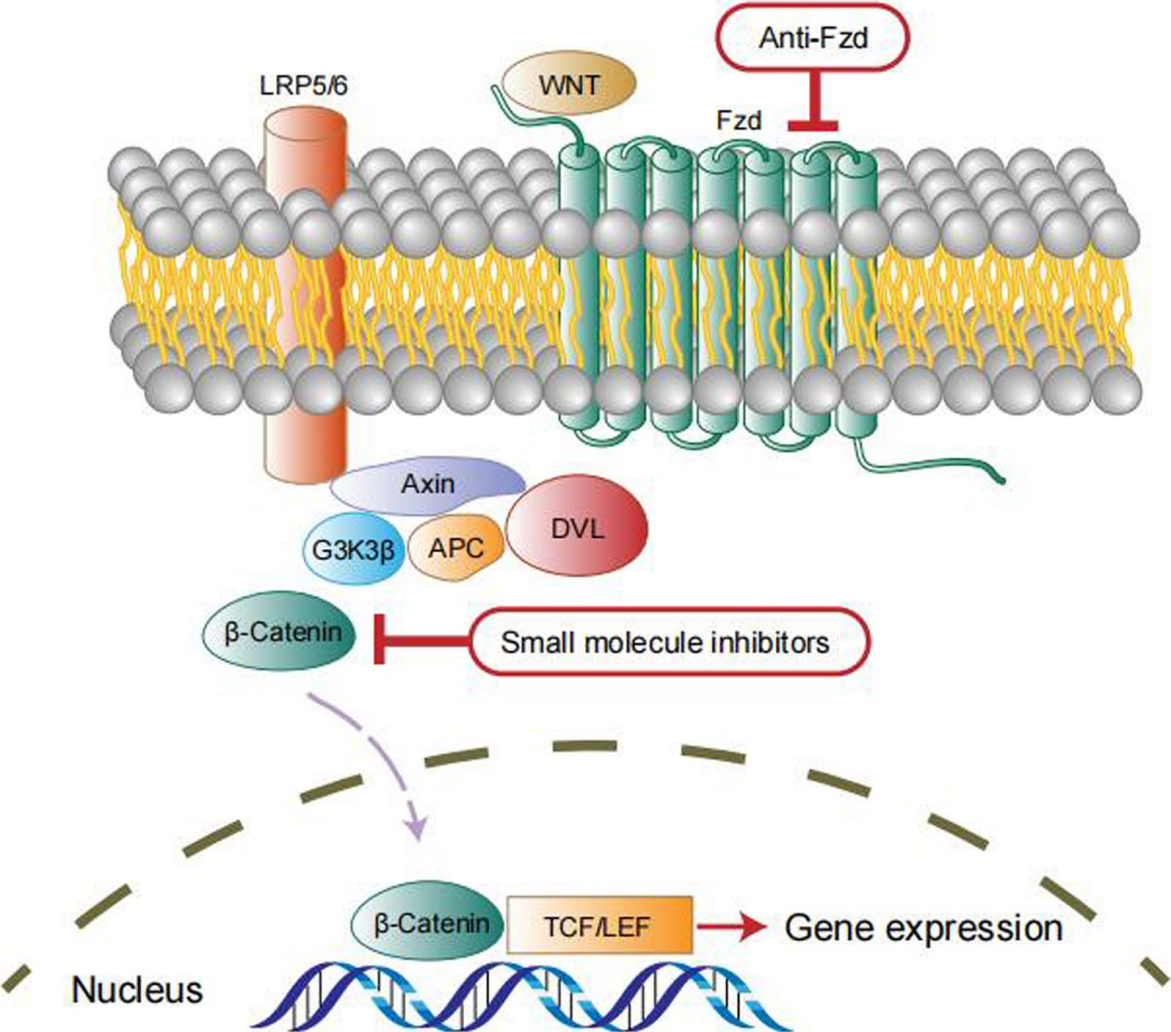
Wnt/β-catenin signaling pathway in OCSCs

**Fig. 5:**
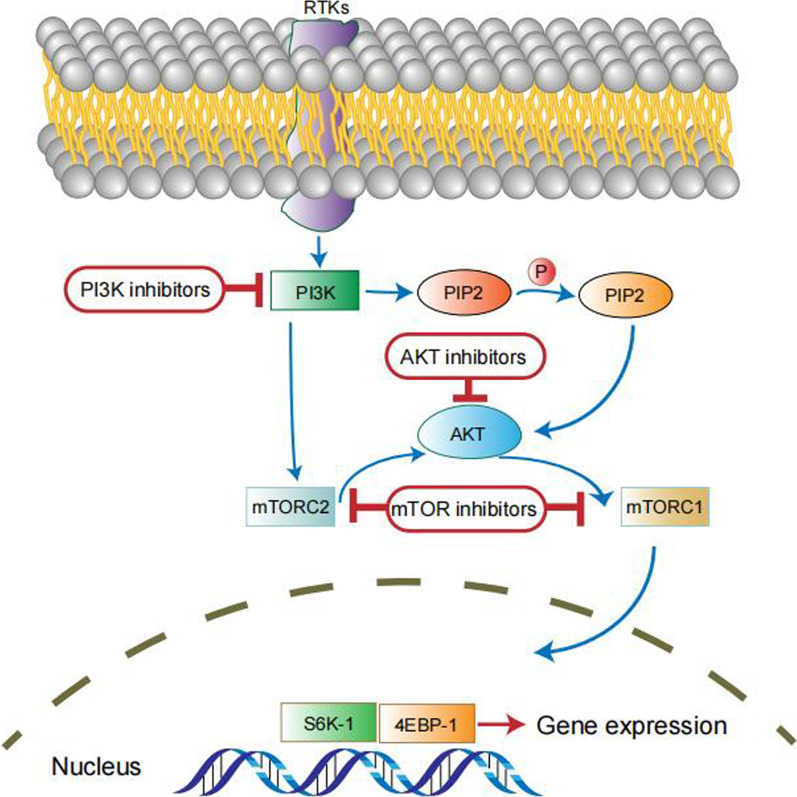
PI3K/AKT/mTOR signaling pathway in OCSCs

**Fig. 6:**
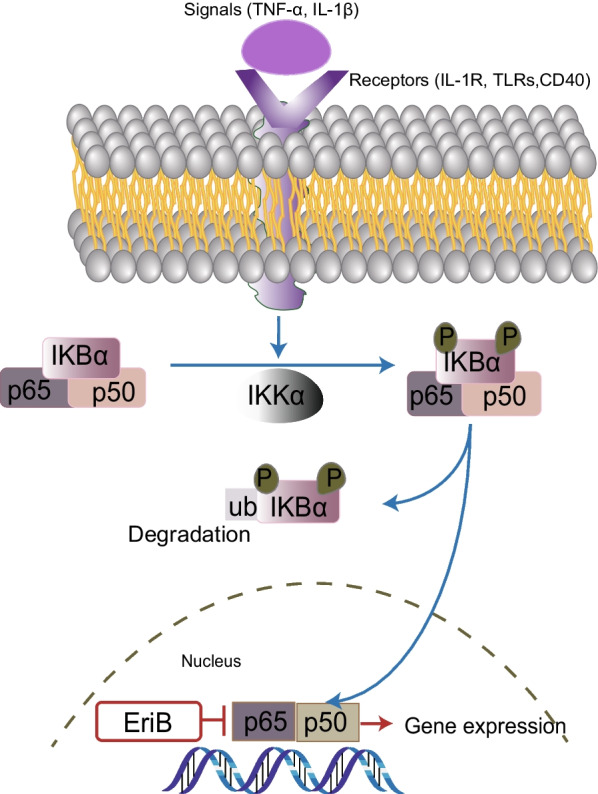
NF-κB signaling pathway in OCSCs

**Fig. 7:**
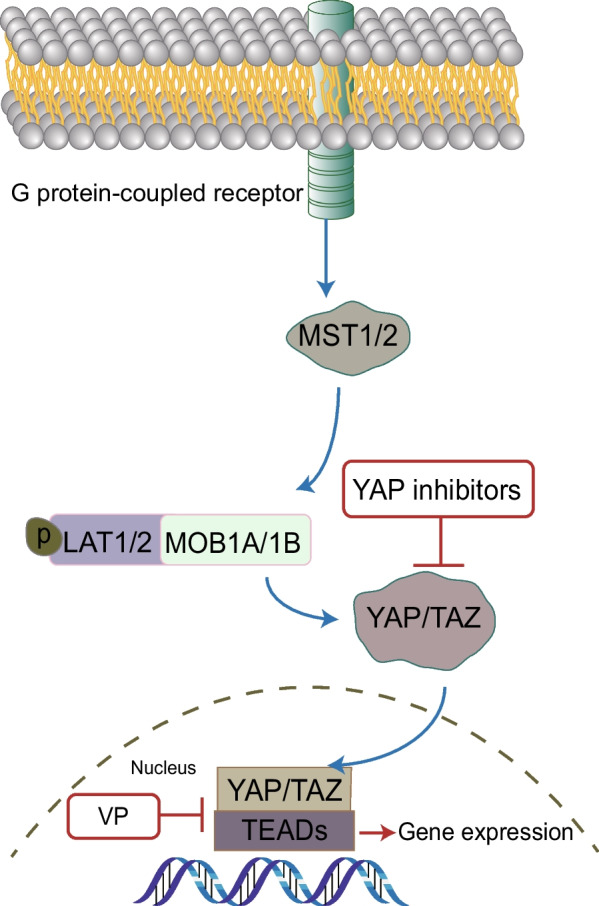
Hippo signaling pathway in OCSCs

A range of signaling pathways were shown to regulate the maintenance, self-replication, differentiation and drug resistance properties of OCSCs. Identification of signaling pathways regulating OCSCs is the key for eradicati–ng OCSCs and in turn harnessing drug resistance and relapse of tumors. We have summarized the related studies as follows and showed the pathways in Figs. 3, 4, 5, 6 and 7.

### Notch signaling pathway

The Notch signaling pathway is one of the vital pathways that constitute the CSC signaling network and is responsible for CSC maintenance, proliferation and differentiation [31]. There are four Notch receptors (Notch 1–4). In particular, the Notch3 signaling pathway is important for OCSC maintenance and tumor resistance to platinum. Notch3 is overexpressed in more than 20% of ovarian serous adenocarcinomas and is related to aggressive subtypes [32]. Notch is a large transmembrane protein that contains an EGF-like extracellular domain that is involved in ligand binding, a transmembrane domain, and an intracellular, nuclear localization domain. After the binding of ligand proteins and Notch receptors, the Notch intracellular domain (NICD) is released by γ-secretase, following the nuclear translocation of NICD to induce transcriptional activation of Notch target genes [33].

There are several Notch pathway inhibitors with different targets and mechanisms of action. γ-Secretase inhibitors (GSIs) are the most classical agents targeting Notch signaling and block the second proteolytic cleavage of Notch receptors and the Notch intracellular domain [34]. Li et al. claimed that inhibition of Notch signaling with a γ-secretase inhibitor markedly inhibits OCSCs from self-renewing and proliferating, significantly downregulates the expression of OCSC-specific surface markers, and reduces the protein and mRNA expression of *Oct4* and *Sox2* in OCSCs [35]. In addition to GSIs, some other compounds that inhibit Notch, such as withaferin A (WFA), have been proven to suppress tumor growth and eliminate CSCs [36]. WFA is a bioactive compound isolated from the plant *Withania somnifera*, and when used in combination with cisplatin, it can prevent the increase of Notch 1, Hes1 and Hey1 expression, which presents a more efficacious therapy for ovarian cancer [36]. Hyeok demonstrated that Galectin-3 maintains OCSCs by activating the Notch1 intracellular domain (NICD1) and leads to an increase in OCSCs and resistance to cisplatin and paclitaxel-induced cell death [37]. Syed et al. proved that adding eugenol to cisplatin-treated ovarian cancer cells synergistically inhibits the Notch-Hes1 pathway and downregulates drug resistance ABC transporter genes, resulting in the inhibition of OCSC growth and elimination of resistant OCSCs [38].

### Wnt/β-catenin signaling pathway

The wingless/integrated (Wnt) signaling pathway is divided into the β-catenin-dependent pathway and noncanonical Wnt pathway. It plays a significant role in cell proliferation, differentiation, migration, and organ development in the period of embryogenesis [39]. Wnt signaling requires a complex combining frizzled (Fz) and lipoprotein receptor-related protein (LRP)-Wnt-induced Fz-LRP6 complex, and complex phosphorylation is a key event in receptor activation, resulting in stabilization of β-catenin. β-Catenin and transcriptional coactivators such as T cell factor (TCF) form complexes that contribute to the transcription of Wnt-responsive genes, inducing cellular responses [40]. Wnt/β-catenin has also been associated with the progression and development of cancer, and it has been identified as one of the most vital pathways involved in CSC maintenance [41]. Accumulated β-catenin activates β-catenin signaling and results in excessive stem cell renewal and proliferation in colorectal cancer [40]. It has been claimed that the Wnt/β-catenin signaling pathway is activated in OCSCs, and targeted inhibition of β-catenin potently sensitizes cells to cisplatin and decreases OCSC sphere formation [42].

Chau et al. claimed that c-kit is a key regulator of OCSC survival and proliferation. Studies have shown that the activation of Wnt/β-catenin and ATP-binding cassette G2 downstream of c-kit lead to the acquisition and maintenance of chemoresistance and the tumor-initiating capacity of ovarian cancer cells, indicating that Wnt/β-catenin combined with c-kit could be significant for improving OC patient prognoses [43]. Several chemical compounds have been proven to exhibit inhibitory effects against OCSCs by suppressing the Wnt/β-catenin signaling pathway. Theaflavin-3,3′-digallate (TF3), a polyphenolic compound extracted from black tea, exhibits anticancer properties by downregulating the protein levels of β-catenin, LEF-1, c-Myc and cyclin D1 in OCSCs. Additionally, the overexpression of β-catenin attenuated the inhibitory effect of TF3 on the viability and tumor sphere formation capacity of OCSCs [44]. Ginsenoside-Rb1, a natural saponin isolated from the rhizome of *Panax quinquefolius* and *notoginseng*, and its metabolite compound K could effectively suppress OCSC self-renewal by downregulating β-catenin/T cell factor-dependent transcription and the expression of its target genes ATP-binding cassette G2 and P-glycoprotein, which is associated with the Wnt/β-catenin pathway [45]. Calcitriol, an active metabolite compound of vitamin D, could deplete the OCSC population and impede the growth of xenograft tumors by inhibiting the Wnt/β-catenin pathway via the VDR pathway [46].

### PI3K/AKT/mTOR signaling pathway

The phosphoinositide 3-kinase (PI3K)/AKT/mammalian target of rapamycin (mTOR) pathway links the response to growth-related hormone receptor interactions to downstream targets, affecting cell proliferation, survival, and apoptosis [47]. The PI3K/AKT/mTOR pathway, found in approximately 70% of ovarian cancers, is associated with aggressive phenotypes, chemoresistance and poor prognosis [48]. PI3K activates AKT/mTOR/p70S6K1 signaling to regulate the G1 cell cycle and apoptosis in ovarian cancer, and inhibition of the PI3K/AKT/mTOR pathway has been found to disrupt ovarian cancer cell proliferation and trigger cell death [49]. Although it has been widely accepted that the PI3K/AKT/mTOR pathway is an attractive therapeutic target for OC treatment, many drugs have not advanced into late-phase clinical studies for several reasons [48].

Deng et al. claimed that the acquisition of EMT and enhancement of CSC marker expression in chemoresistant epithelial ovarian cancer cells were associated with the activation of PI3K/AKT/mTOR signaling [50]. Similar to the two pathways above, several emerging compounds have the potential to be used as novel chemotherapeutic drugs for OC by targeting PI3K/AKT/mTOR signaling. N-t-boc-Daidzein is an isoflavone Daidzein that is capable of inducing apoptosis in EOC cells, including OCSCs, which may be accomplished by promoting the degradation of AKT and inhibiting the mTOR pathway [51]. A newly synthesized genistein derivative, DFOG, has been proven to exert anticancer activity by targeting OCSCs and to reduce OCSC oncogenicity in vitro through inhibitory effects on the PI3K/AKT/mTOR and/or ERK and/or NF-κB pathways [52]. ST6GALNAC1 is responsible for encoding a sialyltransferase that acts as a catalyst for the synthesis of cancer-related sialyl-Tn antigen, which is related to metastasis and poor prognosis in patients with cancer. Wang et al. claimed that ST6GALNAC1 promoted cell proliferation, migration and invasion abilities by activating the PI3K/AKT pathway in OCSCs, providing a novel approach to the study of drug resistance and may contribute to discovering new therapies curing OC [53].

### Other signaling pathways

The Nuclear factor-κ light chain enhancer of activated B cells (NF-κB) is a group of transcription factors involved widely in inflammation, cell growth and apoptosis, including RelA (p65), c-Rel, RelB, p50 (NF-κB 1) and p52 (NF-κB 2) [54]. The NF-κB pathway can be divided into canonical and non-canonical pathway, which may be crucial to the maintenance of the proliferative cell population and CSC populations in tumors, respectively [55]. It has been reported that CD44 + cells promote the stem characteristics of OCSCs by up-regulating the expression of RelA, RelB and IKKα and adjusting the nuclear activation of p50/RelA dimer [56]. The compound Eriocalyxin B (EriB), an analogue of oridonin, has been proved to inhibit NF-κB activity by decreasing the levels of nuclear p65 in the OCSCs, accompanied with the suppression of cytokine production and eventually promoting OCSCs death [57]. 6-phosphofructo-2-kinase/fructose-2,3-biphosphatase 3 (PFKFB3) is vital to the first controlled step in glycolysis which is critical for CSCs. Jiang et al. claimed that PFKFB3 upregulated in CSC-enriched ovarian cancer cells and prompted clonogenicity, sphere-formation in ovarian cancer cells. Furthermore, the over-expression of PFKFB3 has been proved to up-regulate NF-κB activation. The compound 3PO has been proved to inhibit the expression of PFKB3; however, it has not been applied clinically on account of its poor water solubility. Another PFKFB3 inhibitor, PFK158 has been reported to impede tumorigenesis and tumor-initiation in vivo and in vitro, also the first PFKFB3 inhibitor being approved by the U.S. Food and Drug Administrator (FDA) to clinical trial in patients with tumors including ovarian cancer [58].

The Hippo signaling pathway, consisted of a broad range of proteins, is a highly evolutionarily conserved regulator of biological processes, including differentiation, cellular proliferation and cell growth [59]. The core of the Hippo pathway comprises a core kinase cassette that consists of protein kinase MST1/2 and tumor suppressor LSTSA/2. The major targets of the Hippo pathway are Yes-associated protein (YAP) and transcriptional coactivator with PDZ-binding motif (TAZ), whose activity controlled by multiple upstream branches [60]. While plenty of researches show that the activation of YAP and TAZ can induce CSC properties in various malignancies, they can be prevented from interacting with transcriptional enhancer factor TEA domain family (TEADs) for suppressing the transcription of target genes. Xia et al. firstly claimed the self-renewal and chemoresistance properties of OCSCs depend on YAP and TEADS. AKT phosphorylation has been detected in OCSCs followed by YAP/TEADs silenced, indicating that the Hippo pathway may interact with other signaling pathways [61]. Verteporfin (VP), a light-activated drug used in photodynamic therapy for the choroidal neovascular membranes, has been reported as a newly identified YAP inhibitor. Feng et al. reported that VP treatment can prevent the accumulation of YAP in nucleus and the transcriptional coactivator activity of YAP, resulting in the suppression of OC progression [62].

3. Cell surface markers in OCSCs

It is now clear that in the same type of tumor, there are different populations of CSCs defined by one specific marker or different combinations of markers. Identifying and functionally characterizing OCSCs could support the development of effective therapies. Herein, we will discuss several reliable OSCS biomarkers and their relationships to OC chemoresistance and targeting potential for therapies.

### CD44

CD44, a widely expressed surface transmembrane glycoprotein and the main surface receptor with a role in cell–cell interaction, adhesion and migration, is found in many solid tumors, including OC [63]. CD44 was first identified and cloned in 1989 and represents a polymorphic group of surface proteins that range from 80 to 200 kDa in size [64–66]. It was originally identified as a receptor for hyaluronan or hyaluronic acid and later a receptor `to several other ligands, also a marker for cancer stem cells of several types. Several studies have demonstrated the association of CD44 with poor prognosis in EOC patients [67, 68]. More importantly, overexpression of CD44 in OC has been shown to be strongly related to the occurrence of metastasis and disease relapse [69]. Among these studies, the connection between CD44 and CSC maintenance has been emphasized, and CD44 + ovarian cancer cell subpopulations have been proven to initiate tumorigenesis and promote disease recurrence by recapitulating the original tumor [70].

Casagrande et al. demonstrated that OCSCs were characterized as CD44+/MyD88 + cells showing cytokine and chemokine production, high capacity for repair, resistance to conventional chemotherapies, capacity to form spheroids in suspension, and most importantly, ability to recapitulate the original tumor in vivo. In that study, CD44 + OCSCs were capable of expressing high levels of the gene encoding the tight junction protein claudin-4, which was proven to be a high-affinity natural receptor for *Clostridium perfringens* enterotoxin (CPE). Animals with chemotherapy-resistant CD44 + OCSC tumors can gain cure or long-term survival through repeated i.p. injections of CPE. This study supports that CPE-based therapy targeting CD44 + OCSCs may have great potential for chemotherapy-resistant OC patients [71]. MiR-199a was found to specifically regulate CD44, and the overexpression of miR-199a led to reduced expression of CD44, resulting in the inhibition of proliferation, migration and invasion of OCSCs [72].

### CD133

CD133, also known as Prominin-1, is a 97 kDa pentaspan membrane glycoprotein originally found on neuroepithelial stem cells in mice and later in human tissues [73]. The physiologic function of CD133 in normal biology and the progression of cancer remains elusive, and most studies focus on its use as a cell surface marker for the detection of somatic stem cells and CSCs [74]. CD133 alone or in combination with other markers has widely been used to identify stem cells from different kinds of cancers, including brain cancer, prostate cancer and OC. Among these studies, CD133-expressing CSCs show self-renewal potential and the ability to regenerate a histologically similar tumor mass [17, 75, 76]. Although there is no adequate knowledge about the molecular underpinnings of CD133 in cancer, a large range of current studies demonstrate that CD133 plays an important role in predicting overall survival, disease-free survival, and progression-free survival in several kinds of solid cancers [77]. Herein, a spectrum of therapies targeting or relating to CD133 have been developed.

A monoclonal antibody (mAb) against the CD133 fusion protein dCD133KDEL has been found to be effective in targeting CD133 + tumor-initiating cells, which are also CSCs, resulting in a decrease in both OVCAR5 cells and ovarian cancer tumor growth. This novel deimmunized toxin could arrest the proliferation of OC cells in vitro and in vivo, and it can serve as a novel treatment regimen for targeting CD133 + OCSCs [78]. Due to the complex molecular structure of CD133, the effect of CD133 antibody-targeted therapy is still limited. Long et al. utilized the Cre/LoxP regulation system to selectively augment *tBid*, a potent suicide gene, and introduced *tBid* to CD133 + OCSCs, significantly inducing cell apoptosis and inhibiting CD133 + OCSC growth both in vitro and in vivo. In addition, Cre/LoxP system-mediated *tBid* overexpression augmented the cytotoxic effect of cisplatin in CD133 + OCSCs, representing a future clinical approach for preventing OC metastasis and recurrence [79]. Regarding the direct interaction between CSCs and chemokines produced by inflammatory cells, some studies have explored the role of chemokines and cytokines, such as interleukin (IL)-17 and IL-23, in a subpopulation of OCSCs. Xiang et al. demonstrated that IL-17 could promote self-renewal in vitro and enhance the tumorigenic potential of CD133 + OCSCs in xenograft mice through the NF-κB and p38 MAPK signaling pathways [80]. Similarly, Wang et al. indicated that IL-23 and its receptor are both mainly expressed in OCSCs and that IL-23 could promote its self-renewal ability via activation of the STAT3 and NF-κB pathways [81].

### ALDH

Aldehyde dehydrogenase (ALDH) is a family of ubiquitous enzymes located in nearly all mammalian tissues [82]. Several studies have proven that ALDH plays a role in catalyzing the oxidation of aldehydes, subsequently contributing to cellular homeostasis related to the function of stem cells, such as self-renewal capability and stress-resistant properties [83]. ALDH1 is one of the ALDH superfamily of enzymes that converts retinol to retinoic acid and functions as a modulator of cell proliferation, cellular detoxification and stem cell differentiation [84, 85]. It has been proven to be a useful marker for CSCs and is widely used to isolate CSCs in various malignancies, including OC [86, 87]. In OC, ALDH1A1 has been proven to be associated with chemoresistance and may be a potential therapeutic molecular target [88]. However, the mechanisms and signaling pathways of the biological effects of ALDH1 in OCSCs remain unclear, and there remains some controversy about the correlation between ALDH1A1 and OC patient prognosis, which deserves further exploration [89, 90].

Kim et al. indicated that high ALDH1A1, the main isoenzyme of ALDH1, led to NRF2 activation through the p62-associated pathway in ALDH1-high OCSCs, resulting in CSC properties such as drug resistance, colony formation, tumor growth and high stemness marker expression. More importantly, all-trans retinoic acid (ATRA) was proven to have the ability to suppress ALDH1 expression, subsequently suppressing CSC properties in ALDH1-high cancer cells by inhibiting NRF2 activation, which suggests the molecular basis of the ATRA effect in CSCs [91]. Similarly, Young et al. claimed that ATRA can target ALDH1 + OCSCs and suppress sphere formation ability, cell migration and invasion and tumorigenesis via ALDH1/FoxM1/Notch1 signaling [92]. In addition to ATRA, DNA damage-binding protein 2 (DDB2) was proven to suppress non-CSC-to-CSC conversion through repression of ALDH1A1 transcription, limit the CSC subpopulation, and finally halt tumor growth in OC [93]. Kakar et al. claimed that WFA alone or in combination with cisplatin significantly inhibited the spheroid formation of isolated ALDH1 + OCSCs in vitro and reduced its expression in tumors collected from mice bearing orthotopic OC by suppressing the expression of securing, an “oncogene,” which suggested the mechanisms of their antitumor effects [94]. According to several studies [23, 76, 95], ALDH combined with CD133 may be the best supported OCSC marker. Choi et al. found that bone morphologenetic protein 2 (BMP2) could act as a feedback mechanism by promoting the expansion of ALDH + CD133 + OCSCs and restricting the growth of progenitors, which supported BMP2 as a therapeutic target in OC [96].

4. Drugs targeting OCSCs

A new method to find novel therapies for diseases or target molecules for drugs with already existing indications is drug repositioning or repurposing, which may drastically save time and other resources compared with traditional drug development. There are also some well-known drugs targeting OCSCs, showing a potential way to treat OC.

### Metformin

Metformin, a biguanide derivative, is considered a first-line antidiabetic drug for managing type 2 diabetes. A range of studies have indicated that metformin may improve lipid metabolism and weight loss, reduce cardiovascular incidence, inhibit brain function, slow cognitive decline, and reduce the risk of dementia [97–99]. More importantly, several studies have proven that metformin has antitumor effects and improves chemotherapy sensitivity [100, 101]. Epidemiological studies have indicated that in many types of cancer, the cancer-related mortality rate of patients who take metformin is significantly lower, including pancreatic, breast, liver and endometrial cancers [102]. In OC, studies have proven that metformin has anti-proliferative and pro-apoptotic effects on cancer in vitro and in vivo. It has also been demonstrated that a major mechanism for metformin’s ability to inhibit OC growth is metformin impacting OCSC growth.

Shank et al. reported that metformin was active against primary human OCSCs in vitro and that metformin therapy alone slowed the growth of OCSCs in vivo [103]. Based on the theory that CSCs and tumor vasculature may be mutually dependent on each other, they demonstrated that the antiangiogenic effects of metformin are due to the actions of metformin on CSCs [103, 104]. Interestingly, they also mentioned earlier studies indicating that metformin acted primarily on tumors with p53 mutations, while 95% OC carries p53 mutations [105]. This study indicated the mechanisms of metformin’s antitumor effect and provided evidence of its potential significance for OC patients. Zhang et al. showed that low concentrations of metformin selectively inhibited CD44 + CD117 + OCSCs via downregulation of epithelial mesenchymal transformation [106]. They also illustrated that metformin inhibited tumor sphere formation, decreased SKOV3 and primary OC growth, and most importantly, enhanced the anticancer effect of cisplatin, which may be effective in preventing OC recurrence and improving patients’ long-term survival.

### Salinomycin

Salinomycin (Sal), a monocarboxylic polyether antibiotic naturally isolated from *Streptomyces albus* and initially used as an agricultural antimicrobial agent [107], acts as an ionophore and promotes the transfer of cations across biological membranes via an exchange diffusion mechanism [107]. Studies have proven that Sal selectively kills CSCs in some types of cancer, such as leukemia, breast cancer, colorectal cancer, lung cancer, and gastric cancer, but the underlying mechanisms have not been well elucidated [107–112]. It has been well accepted that paclitaxel combined with Sal can produce strong antitumor effects for the eradication of breast cancer and CSCs, which provides inspiration for the application of Sal in OC [113]. It has been demonstrated that Sal inhibits growth and induces apoptosis in the cisplatin-resistant human OC cell line C13 in vitro and exhibits significant efficacy in vivo, suggesting that Sal is a promising antitumor agent in cisplatin-resistant OC therapy. There are no adequate studies about the activity against OCSCs, which demands deep exploration.

*SOX2* has been recognized as a cancer stem cell-associated gene, and the overexpression of *SOX2* is related to cell proliferation, cell migration, resistance to cisplatin treatment and tumorigenicity of ovarian cancer cells [114]. Lee et al. demonstrated that paclitaxel combined with Sal silenced SOX2 expression and increased apoptosis of OCSCs, while the underlying mechanism required further investigation [115]. In addition to combining Sal with paclitaxel, Sal can also be utilized with other CSC markers to improve therapeutic efficiency. Mi et al. showed that Sal exerted significantly increased cytotoxic effects in CD133 + OC cells and reduced the CSC percentage in OC cells [116]. Due to the poor water solubility of Sal, Sal-loaded antibody-conjugated nanoparticles have been developed for their delivery to CSCs. They utilized CD133-SAL-NPs to improve the targeting of Sal toward OCSCs, resulting in an increased cytotoxic effect, which represented a promising approach for the treatment of OC.

### Calcium channel blockers (CCBs)

Calcium is a significant signal transduction element involved in many different cell processes, such as proliferation, differentiation, growth, cell death and apoptosis [117]. In tumor cells, it is well known that calcium channels play important roles in tumorigenesis and tumor progression by controlling gene expression, DNA synthesis, cell cycle progression, apoptosis, proliferation and migration [118–120]. The importance of calcium channels in tumorigenesis and tumor progression shows the possibility of targeting calcium channels during tumorigenesis. Studies proved that in glioblastoma stem cells, calcium channels and signaling pathways were enriched, which elicited vital cell functions in response to extracellular cues [121]. Through a screen of 72 ion channel blockers, there were 10 drugs acting on calcium-related channels among the 12 drugs capable of reducing glioblastoma stem cells [122]. In OC, the calcium channels involved in promoting cancer behaviors are mostly voltage-gated calcium channels, nonvoltage-activated calcium channels and intracellular calcium channels, such as the IP_3_R and Orai families.

Lee et al. established a high-throughput screening system to identify agents that inhibit the sphere-forming property and proliferation of CSCs and screened approximately 1000 compounds [123]. They found that four calcium channel blockers had anticancer effects against OCSCs, mediated via L- or T-type calcium channels. These four CCBs induced OCSC apoptosis via inhibition of AKT and ERK signaling; more importantly, the combination treatment with CCBs and cisplatin enhanced drug sensitivity in a CSC-enriched epithelial OC population. Similarly, CCBs combined with poziotnib, a panepithermal growth factor receptor inhibitor, could inhibit the expression of stem cell markers, especially CD133, NANOG, and KLF4, and could suppress the phosphorylation of STAT5, AKT and ERK, a process involved in the self-renewal ability of OCSCs [124].

5. Challenges and future

Chemoresistance remains one of the major challenges for OC patients. There are various molecular mechanisms involved in chemoresistance, such as decreasing drug concentrations by drug efflux pumps, degrading drugs by detoxifying enzymes, which are related to the inherent ability of cancer cells to survive chemotherapy. The acquired chemoresistance ability of cancer cells is related to the mutation and expression of genes, which is developed through natural selection of changes to gain survival advantages [125]. p53 is known as tumor-suppressed gene and barrier to CSCs formation [126]. It is known that most of HGSOC harbor mutant p53 status, which enhances metastasis and chemoresistance. Accumulated data suggest that the chemoresistant property of CSCs interweave with mutant p53 status [127]. Ramraj et al. developed an p53-targeted strategy, mainly aimed at OC cells that harbor mutant p53, for maintenance therapy of HGSOC [128]. The contribution and mechanisms of OCSCs inducing cancer chemoresistance and recurrence need further discussion, which may effectively eradicate OC. Another great challenge is the isolation of OCSCs. Although the most common method to isolate OCSCs is to select for cells by the expression of markers, already discussed above, it is quite reliable. It may be more reliable to classify OCSC populations by transcriptional profiling considering the inherent heterogeneity of OC cells.

## Conclusion

CSCs are associated with the entire process of ovarian cancer development, including initiation, metastatic progression, therapeutic resistance or disease recurrence. To overcome cancer metastasis, new strategies are still needed in current therapy. Despite our increasing knowledge of the traits of OCSCs, there remains much that we do not know. As we delve more into tumor biology and the mechanisms regulating OCSCs, there is a greater understanding of the strategies that may help to find reliable strategies for OC therapy. Our review has shown that there are different therapies to eliminate OCSCs in different ways. Findings from such a review could assist researchers in exploring targeted therapy against OCSCs, which will improve patient survival and decrease tumor relapse among OC patients.

## Data Availability

The datasets used and/or analyzed during the current study are available from the corresponding author on reasonable request.

## Abbreviations

OC: Ovarian cancer
CSCs: Cancer stem cells
OCSCs: Ovarian cancer stem cells
NICD: The Notch intracellular domain
GSIs: γ-Secretase inhibitors
WFA: Withaferin A
NICD1: The Notch1 intracellular domain
Wnt: Wingless/integrated
Fz: Frizzled
LRP: Lipoprotein receptor-related protein
TCF: T cell factor
TF3: Theaflavin-3,3′-digallate
PI3K: Phosphoinositide 3-kinase
mTOR: Mammalian target of rapamycin
CPE: *Clostridium perfringens* Enterotoxin
mAb: Monoclonal antibody
ALDH: Aldehyde dehydrogenase
ATRA: All-trans retinoic acid
DDB2: DNA damage-binding protein 2
BMP2: Bone morphologenetic protein 2
Sal: Salinomycin
CCBs: Calcium channel blockers
